# Role of scalp hypothermia in patients undergoing minimally invasive evacuation of hypertensive cerebral hemorrhage

**DOI:** 10.12669/pjms.35.5.593

**Published:** 2019

**Authors:** Yueling Zhang, Ling Song, Jianfen Zhao

**Affiliations:** 1 Yueling Zhang, Department of Operating Theatre Binzhou People’s Hospital, Shandong, 256610, China; 2 Ling Song, Department of Cardiothoracic Surgery, Binzhou People’s Hospital, Shandong, 256610, China; 3 Jianfen Zhao, Department of Cardiovascular, Binzhou People’s Hospital, Shandong, 256610, China

**Keywords:** Hypertensive cerebral hemorrhage, Mild hypothermia, Micro-traumatic evacuation of cerebral hemorrhage

## Abstract

**Objective:**

Hypertensive intracerebral hemorrhage (HICH) is one of the common multiple diseases in neurology. Patients with severe HICH have high risk of disability and poor prognosis.

**Methods:**

In order to explore the clinical effect of mild hypothermia combined with micro-traumatic evacuation of cerebral hemorrhage in the treatment of severe HICH, 136 patients with severe HICH were selected and divided into control group and study group using random number table method, 68 each group. The control group was treated with micro-traumatic evacuation of cerebral hemorrhage on the basis of conventional symptomatic treatment, while the study group was treated with mild hypothermia combined with micro-traumatic evacuation of cerebral hemorrhage on the basis of conventional symptomatic treatment. After treatment, the two groups were followed up for eight weeks.

**Results:**

The overall effective rate, residual hematoma volume, rebleeding rate, National Institute of Health stroke scale (NIHSS) score, Barthel index score and incidence of adverse reactions after treatment were observed and compared. The overall effective rate of the study group was 89.7%, which was significantly higher than that of the control group (67.6%). The mortality rate of the study group was 3.0%, which was significantly lower than that of the control group (14.7%, P<0.05). The residual hematoma volume and rebleeding rate of the study group were significantly lower than those of the control group (P<0.05). Before treatment, the NIHSS score and Barthel index score of the two groups had no significant differences (P>0.05). After treatment, they were improved, and the improvement of the study group was more significant (P<0.05). The incidence of adverse reactions in the study group was 10.0%, which was significantly lower than that in the control group (36.0%, P<0.05).

**Conclusion:**

Mild hypothermia in combination with micro-traumatic evacuation of cerebral hemorrhage has significant clinical effect in the treatment of severe HICH. It can significantly improve neurological function and quality of life, causing few adverse reactions. Its clinical application value is high.

## INTRODUCTION

Hypertensives intracerebral hemorrhage (HICH), one of the common complications of hypertension, is a phenomenon of severe hemorrhage induced by vascular rupture in brain tissues caused by excitement, excessive exercise and brain work. When HICH happens, the amount of brain bleeding is usually above 50 mL. HICH has a high mortality and disability rate in clinical practice.^1^,^2^ The clinical manifestations of HICH include nausea and vomiting, headache, restlessness, lethargy, coma, etc. If the patients were not hospitalized in time or treated improperly, the mortality rate will be extremely high.^3^ At present; there is no specific treatment for HICH. In the past, craniotomy evacuation of hematoma was often used, but the trauma was large and the incidence of complications was high.^4^ Micro-traumatic evacuation of cerebral hemorrhage is one of the new surgical methods developed in recent years. It has advantages of short operation time, less intraoperative blood loss and quick recovery.^5^ In addition, a study has shown that mild hypothermia therapy can effectively improve brain cell metabolism,^6^ promote lactic acid decomposition, reduce brain oxygen consumption, reduce blood-brain barrier permeability, and thus effectively protect brain tissue and nerve cells.

In order to find a more reasonable and effective treatment plan for severe HICH and improve the survival rate and quality of life of patients, the author applied mild hypothermia combined with micro-traumatic evacuation of cerebral hemorrhage in the treatment of severe HICH patients, compared them with controls who received micro-traumatic evacuation of cerebral hemorrhage.

## METHODS

One hundred and thirty-six patients with severe HICH were selected from patients who were admitted to our hospital between August 2016 and August 2018. All of them satisfied the following inclusive criteria: satisfying relevant diagnostic criteria of severe HICH described in Consensus of Chinese Experts on Endovascular Therapy for Acute Ischemic Stroke and being diagnosed as severe HICH by computed tomography (CT).^7^ Exclusion criteria included having severe impairment in organs such as the heart, liver, kidney and lung, having poor treatment compliance and drug tolerance, having allergic constitution and drug allergy, having contraindications to early calvarial mild hypothermia treatment and micro-traumatic evacuation of cerebral hemorrhage, having congenital coagulation dysfunction and taking drugs which could affect coagulation function one month before admission, and having mental and consciousness disorders. The patients were divided into a study group and a control group using random number table, 68 each group. There were 42 males and 26 females in the control group, with an average age of (73.01±5.45) years (50~86 years) and an average interval between onset and admission of 3.23±0.52 hours (1~5 hours). There were 39 males and 29 females in the study group, with an average age of (72.18±5.24) years (46~88 years) and an average interval between onset and admission of (3.42±0.71) hours (0.5~7 hours). The baseline characteristics including average age, sex and time between onset and admission of the two groups were similar, and the difference was not statistically significant (P>0.05); hence the results of the two groups could be comparable. The research project was approved by the ethics committee of our hospital. All patients who participated in the study have signed consent.

### Treatment Methods

Two groups of patients were treated with blood pressure control, prevention of complications and dehydration. The control group was treated with micro-traumatic evacuation of cerebral hemorrhage. The parameters of the hematoma sites were positioned by CT. After conventional skin disinfection, local anesthesia was performed. The point with the widest hemorrhage on the CT plane was taken as the puncture point, avoiding the important functional areas. Drilling was performed after the intracranial hematoma puncture needle (model: YL-1; manufacturer: Beijing Wanteford Company, China) was connected to the electric drill. After penetrating the dura mater, the electric drill and needle core were removed, and the drainage tube with plastic needle core was inserted into the hematoma cavity. Hematoma aspiration was performed. Hematoma pulverizer was connected. 4 mL of flushing fluid was injected into the hematoma, if the blood volume in the drainage liquid was 70% that of the bleeding amount, then the surgery was considered as successful. Then the hematoma pulverizer and drainage tube were removed. The surgical site was bound up using aseptic dressing.

The study group was given mild hypothermia treatment on the basis of the above treatment, and the specific measures were as follows. ZLJ-2000 comprehensive therapy instrument for brain cooling rescue (brand: Minkang; model number: ZLJ-2000; product code: mk105779; manufacturer: Wuhan Minkang Medical Devices Co., Ltd., China) was selected, and the probe was fixed on the head. Omron E5AK thermometer (brand: Omron; model number: E5AK-AA2-500; manufacturer: Omron Corporation, Japan) was used to detect the pericranial temperature of patients, and the scalp temperature was maintained at 33-35 °C. After five days of continuous treatment, the temperature was recovered.

The two groups were treated strictly following the treatment plan during the treatment period and were given comprehensive nursing after the operation. The specific measures are as follows. Position nursing care was performed firstly. In posture nursing, hemiplegic patients were paid attention to. The hemiplegic limbs were moved to the corresponding nursing position, and limbs were reasonably placed to prevent occurrence of joint deformity. Before changing the position, the correct posture was kept at all times. The lateral position was the main posture, which could stimulate the nerve and perception of patients. Such a nursing method could help targeted treatment and improve nursing effect. Next was drainage tube nursing. After minimally invasive surgery, patients with severe HICH sterile drainage bags were connected via the hematoma cavity drainage tube. Nurses fixed the drainage bag at a position less than 20 cm above the patient’s head, but if it was a ventricular drainage tube, the drainage bag should be fixed at a position which was 10-15 cm above the patient’s incision. The drainage bag was replaced strictly according to aseptic operation. The changes of the nature, color and volume of drainage fluid were observed, and attending physicians were informed if there were any changes. The last one was nutritional support. Patients with severe HICH usually presented a state of high decomposition and high metabolism after operation, and energy consumption increased significantly. Moreover high protein catabolism could lead to negative nitrogen balance in patients, which might increase mortality and disability rates significantly. In view of such a condition, nurses provided patients with nutritional support to accelerate the reconstruction of brain tissue functions. 10 g/d albumin and 500 ml/d amino acid were intravenously infused, and high-protein, high-calorie, high-vitamin and digestible fluids were given to patients by means of nasal feeding from the 3rd day after operation. After treatment, the two groups of patients were followed up for 8 weeks.

Patients were considered as cured if admission symptoms and vital signs returned back to the normal ranges and all examinations results were normal. If admission symptoms and vital signs were significantly relieved and all examination results were basically normal, then the treatment was considered as significantly effective. If admission symptoms and vital signs were alleviated and all examination results improved, the treatment was evaluated as effective. If admission symptoms, vital signs and examination results were not significantly improved, the treatment was thought as ineffective. The overall effective rate could be calculated using the formula: the overall effective rate = (number of cured cases + number of significantly effective cases + number of effective cases)/total number of cases × 100 %.

The residual hematoma volume and rehaemorrhage rate of the two groups were observed. The NIHSS score of the two groups was evaluated. The higher the NIHSS score, the more serious the neurologic impairment. Activities of daily living were evaluated using Barthel index; the score was between 0 point and 100 points. The higher the Barthel index score, the better the activities of daily living. The incidence of adverse reactions of the two groups was observed.

Data were statistically analyzed using SPSS ver. 23.0. Measurement data were expressed as Mean±SD and processed by t test. Enumeration data were expressed as n (%) and processed by Chi-square test. Difference was considered as statistically significant if P<0.05.

## RESULTS

The overall effective rate of the study group was 89.7%, which was significantly higher than that of the control group (67.6%) (P<0.05); the mortality rate of the study group was 3.0%, which was significantly lower than that of the control group (14.7%) (P<0.05, Table-I).

**Table I:** Clinical overall effective rate between the two groups.

Group	Study group (n=68)	Control group (n=68)	X^2^	P
Cured	16	8	/	/
Significantly effective	11	12		
Effective	34	26		
Ineffective	5	12		
Death	2(3.0%)	10(14.7%)	4.887	<0.05
Overall effective rate	61(89.7%)	46(67.6%)	7.294	<0.05

The residual hematoma volume and rehaemorrhage rate of the study group of the study group were significantly lower than those of the control group, and the differences had statistical significance (P<0.05, Table-II).

**Table II:** Comparison of residual hematoma volume and rehaemorrhage rate between the two groups.

Group	Study group (n=68)	Control group (n=68)	t/X^2^	P
Residual hematoma volume (mL)	9.6±3.6	26.1±4.4	21.057	<0.05
Rehaemorrhage rate	10(14.7%)	24(35.3%)	6.435	<0.05

Before treatment, there was no significant difference in the NIHSS score and Barthel index score between the two groups (P>0.05); the NIHSS score and Barthel index score of the two groups after treatment were significantly better than those before treatment, and the improvement of the two indexes in the study group was significant than that in the control group (P<0.05, Table-III).

**Table III:** Comparison of QLQ-C30 scale score between two groups (point, Mean ± SD).

Group		Study group (n=68)	Control group (n=68)
NIHSS score	Before treatment	11.62±2.54	11.81±2.73
	After treatment	4.62±1.51*^#^	6.31±1.43*
Barthel index score	Before treatment	50.87±2.36	50.92±2.41
	After treatment	85.15±5.26*^#^	78.25±5.10*

* ***Note:***: P<0.05 comparing to before treatment;

# P<0.05 comparing to the control group.

In the study group, there were one case of stress ulcer, two cases of intracranial infection, 3 cases of cerebral hernia and one case of multiple organ failure, with an incidence of 10.3%. In the control group, there were six cases of stress ulcer, seven cases of intracranial infection, three cases of cerebral hernia and eight cases of multiple organ failure, with an incidence of 35.3%. The incidence of complications in the study group was significantly lower than that in the control group (X^2^=10.321, P<0.05).

## DISCUSSION

Most patients with severe HICH have coma and serious neurological deficits when they are admitted to hospital. Severe HICH is related to the compression of primary hematoma, the release of blood components in hematoma and the destruction of deep brain structure, and it can lead to hyperpyrexia, endocrine dysfunction and abnormal circulation and respiratory function, which can endanger the life safety of patients.^8^

Micro-traumatic evacuation of intracranial hematoma is a common method in the treatment of HICH. It can quickly remove hematoma and has a definite curative effect. Wang et al. have reported the significant effect of early mild hypothermia treatment on acute severe cerebral hemorrhage.^9^ In this study, curative effects of micro-traumatic evacuation of intracranial hematoma and mild hypothermia in combination with micro-traumatic evacuation of intracranial hematoma were compared and analyzed. The results showed that the overall effective rate of the study group was 89.7%, which was significantly higher than that of the control group (67.6%), the mortality rate of the study group was 3.0%, which was significantly lower than that of the control group (14.7%), indicating that mild hypothermia combined with micro-traumatic evacuation of intracranial hematoma had significant effect in the treatment of severe HICH. In addition, the residual hematoma volume and rehaemorrhage rate in the study group were significantly lower than those in the control group, indicating that mild hypothermia in combination with micro-traumatic evacuation of intracranial hematoma had a significant effect on the elimination of hematoma. The NIHSS score and Barthel index score of the study group were significantly better than those of the control group after treatment, which showed that mild hypothermia in combination with micro-traumatic evacuation of intracranial hematoma could effectively improve the neurological function of patients with severe HICH and quality of life, and it was similar to the results reported by Takeuchi et al.^10^ In terms of safety, studies also confirm that mild hypothermia can significantly reduce the degree of brain edema and intracranial pressure in the treatment of patients with clinical cerebral hemorrhage,^11^,^12^ as well as inhibit the production of oxygen free radicals and neuronal apoptosis. The results of this study also confirm that point as the incidence of adverse reactions in the study group was significantly lower than that in the control group. In conclusion, micro-traumatic evacuation of intracranial hematoma combined with mild hypothermia can effectively improve the curative effect and reduce the incidence of complications in patients with severe HICH, which is worth clinical promotion.

### Authors’ Contribution

**YLZ:** Study design, data collection and analysis.

**LS & JFZ:** Manuscript preparation, drafting and revising.

**YLZ & JFZ:** Review and final approval of manuscript.
